# Necrology

**Published:** 1886-02

**Authors:** Eugene Foster

**Affiliations:** Augusta, Ga.


### Necrology.

### IN MEMORIAM.

### L. A. DUGAS, M. D., LL. D.

A few days ago I was requested by the chairman of your Committee on Necrology to write the memorial tribute to my much beloved and venerated friend, Dr. L. A. Dugas. In fulfilling the task assigned me, it is no part of my purpose or desire to attempt anything like fulsome laudation of the dead. Nor is this tribute presented with the hope of adding to the fame of him whose life was devoted to active, fruitful industry in usefulness to his fellow-man, governed by a single-mindedness to truth, and unswerving fidelity to the discharge of every duty pertaining to his exalted position as a citizen, a much beloved and highly accomplished physician, a genuine philanthropist, and a learned scientist. Dr. Dugas' fame is written in the annals of scientific medicine, and though dead he yet lives and exerts an influence upon men who are members of that profession which he did so much to advance and adorn. The purpose of this biography, as all biographies should be, is for instruction of the living, to inquire into his career to eminence and fame, and to explore the foundation on which he builded so wisely and grandly. In him was a rare type of exalted manhood, and the study of his life should inspire the young men of our profession to emulate his character that they may participate in the royal honors which crown the work of the man who devotes himself to the advancement of his race.

"Louis Alexander Dugas was born January 3, 18c6, in Washington, Wilkes county, Ga., and was the son of Louis Rene Adrien Dugas de Vallon. The De Vallons were of French West Indian descent, immigrating from France some two generations ago to St. Domingo, where they became wealthy planters. His father, although born in St. Domingo, resided constantly in Paris, where his ample fortune enabled him to gratify his literary tastes; he was a gentleman of large and varied information, and had graduated in the law, besides acquiring great proficiency in the sciences. His mother, Mary Pauline Bellumean de la Vincendiere, was a native of St. Domingo, where her parents had been wealthy planters for generations, but always educating their children in Paris, and it was there she met M. de Vallon and married him in August, 1790. A gentleman of leisure and cultivated tastes—one of the old regime—had few inducements to remain in France in those troublous times, and accordingly M. de Vallon and his wife left for St. Domingo to settle on their plantation, where generations of their ancestors had preceded them. They had not been long there, however, before the revolution in the parent country extended itself to the colony, and resulted in the emancipation of the blacks, driving them, with an infant daughter and but slender pecuniary means, to seek refuge in the United States. They landed in Charleston in 1791, and in deference to the republican simplicity of the land of their adoption, dropped the 'de Vallon' from their name and were henceforth simply Dugas.

"After about a year's residence in Georgetown, S. C., they removed to Newport, R. I., where they remained till 1801, when they removed to Fredericktown, Md. Three years in Maryland caused them to remove to Savannah, seeking a warmer climate, and in 1804 they settled in Washington, Wilkes county, where Dr. L. A. Dugas was born, with a twin brother, Louis Charles, who was afterward a planter, and died in 1866. His father died in 1807, and in December, 1810, his mother, a most estimable and accomplished lady, removed to Augusta, established a female seminary, and was so successful as to educate her family and accumulate a competency. His mother educated her son until he was fifteen, with the exception of two or three quarters at the Academy of Richmond county. She had the proud satisfaction of seeing the prosperity of her children, and died in 1854, being then eighty-three years of age.

"Dr. Dugas, in 1820, entered the office of Dr. Charles Lambert De Beauregard, a French emigre', to study medicine. Dr. Beauregard dying in 1822, he entered the office of Dr. John Dent and studied for two years. He then attended lectures in Maryland and Philadelphia, and was graduated at the University of Maryland in March, 1827. The medical department of that University was then considered the best school in the country. He attended all the hospitals of the cities, and feeling the great responsibility of the practitioner he resolved to perfect himself in the European schools. After some plantation practice and study in Georgia, he sailed, in 1828, for Europe, where he remained upward of three years, during which he made himself thoroughly acquainted with England, France, Switzerland, Germany and Italy, but making Paris his headquarters. There he devoted himself with persistent energies to his medical studies, devoting sixteen hours of each day to different branches of science, and so methodically arranging his time, alternating the severer with the more attractive branches of study, as to insure constant interest and avoid weariness.

During his sojourn abroad he began those habits of systematic, varied and diligent study which characterized him during his whole life. Thus we find him devoting the mornings to visiting hospitals and following the professors in their rounds through the wards, visiting patients, and making surgical operations, and attending the dead-house, closely watching post mortem examinations. In the evenings he attended the full courses of lectures at the Sarboune, of such talented professors as Gay Lussac and Thenard on Chemistry, Pouillet on Physics, Arago on Astronomy, Cuvier, Blainville and Geoffrey St. Hilaire on Natural History, Beaumont on Geology, Magendie on Physiology, Boyet, Roux and Velpeau on Surgery, Dupuytren and Lesfranc on Chemical Surgery, Guersent on the Diseases of Children, Cousin on Philosophy, Villemain on Eloquence and Criticism, and Guizot on the History of Civilization. He also attended the lectures of such illustrious men as Baron Larrey, Dubois, Alibert, Biett, Lugol, Broussais, Audral, Louis, Chomel, and Orfila. Having graduated from an American college whose diploma was recognized in Europe he, upon exhibiting his diploma, had free access to all the medical colleges and hospitals. It was during his sojourn in Paris that he observed the revolution from Broussais' deteriorating treatment to the conservative plan of Audral and Louis, and he became an ardent supporter of the latter system. He also studied with interest Civiale's method of Lithotrity, and sucessfully performed this operation four times after his return to America. The writer witnessed one of these operations in the hands of Dr. Dugas, and was charmed by the dexterity, success and coolness of the operator.

With a mind stored with the useful and marvelous education which he had acquired in Europe, he set sail for America and returned to Augusta in June, 1831, and began the practice of medicine. In 1832 he, with Drs. Milton Anthony, Lewis D. Ford, John Dent, Paul F. Eve, and Joseph A. Eve, founded and organized the Medical College of Georgia (now the Medical Department of the University of Georgia.) Dr. Dugas was elected Professor of Anatomy and Physiology ; a few years subsequently he relinquished the chair of Anatomy to Dr. George M. Newton, and became Professor of Physiology and Pathological Anatomy. This professorship he held until 1855, when he was elected to that of Principles and Practice of Surgery, which chair he held until his resignation from the faculty in 1880.

In 1834 Dr. Dugas again visited Europe, having been sent by the Medical College of Georgia with $6.000, to purchase a library and museum for that institution. Having an acquaintance with Parisian collectors he was enabled to purchase a fine museum, with many rare specimens, and a valuable library, embracing many rare books. During this visit to Paris he was elected a member of the Geological Society of France. He returned to Augusta in the fall of 1834, and began the general practice of medicine, devoting more special attention to surgery. In 1851 he again visited Europe and traveled extensively over the continent, visiting the World's Fair at the Crystal Palace in Hyde Park, London. In 1851 he became editor of the Southern Medical and Surgical Journal, published in Augusta. During his editorial management, which lasted for seven years, this journal was noted for its high order of medical journalism, and continued numerous and voluminous contributions from the pen of its illustrious editor.

Dr. Dugas was a medical philosopher. His genius was of that high and rare order which qualified him for thorough investigation and elucidation of abstruse questions in all the multiform departments of medicine. A mere glance at the list of his contributions to medical literature will show the versatility of his genius. Most of these papers are to be found in the volumes of the Southern Medical and Surgical Journal. The remainder appear in the New Orleans Medical and Surgical Journal, the ATLANTA MEDICAL AND SURGICAL JOURNAL, the Transactions of the American Medical Association, Transactions of the Medical Association of Georgia and Transactions of the International Medical Congress. The following papers were from his pen:

Remarks on Pathology and Treatment of Rheumatism. Review of Gerhard on Diseases of the Chest. Purulent Ophthalmia of Infants. Review of Cruveilhier's Pathological Anatomy. Peachleaf in Pertussis. Cases from Note Book. Pathology of Idiopathic or Spontaneous Mortification. Structure of the Urethra. Colica Pictonum. Review of Ammusat's Lectures. Hydrocephulus, Tapping of. Sinapisms to Mammæ in Amenorrhœa. Some of the Causes of Impeded Respiration. Compilation and Review of the Mortuary Statistics of Augusta. On the Value of Pessaries. Review of a Work Attributed to Hippocrates. The Pathology and Treatment of Bilious Fevers. Pathology and Treatment of Convulsions. Report of Surgical Operations on the Eye. Report on the Sigamentum Dentis. Report of Surgical Cases, Amputation of Scirrous Mamma, Amputation of Thigh, Ligation of Brachial Artery, Extraction of Foreign Bodies in Oesophagus, etc. Views on the Origin of Intestinal Worms. Premature Parturition. Outlines of the Anatomy and Pathology of the Liver. Surgical Operations on Patients During Mesmeric Insensibility, with Cases. 4 Papers on this Subject. Review of Dickson's Practice of Medicine. Review of Budd on Diseases of the Liver. Remarks on Tumors of the Neck, with Cases. Notice of Bonnet on Joints. The Use of Quinine in Int. and Remittent Fever. Glanders in the Human Subject, with Case of. Plagiarism of a Medical Author. Strictures on Medical College Circulars. Malformation of the Genito-Urinary

Apparatus. Review of Mitchel on Cryptogamous Origin of Fevers, (and other works.) A Clinical Lecture on Syphilis. A Clinical Lecture on Colic. Remarks on the Treatment of Fractures. Notice of Carpenter's Physiology. Use of Quinine in Singultus and Tetanus. Review of Drake on Diseases of Mississippi Valley. Remarks on Gangrenopsis. Remarks on Dislocation of Radius and Ulna Backwards, with Cases. Diseases of the Uterus, editorial. Chloroform, Ergot, etc., editorial. Lithotrity for Urinary Calcus, with Cases. Progressive Muscular Atrophy. Diet, Drink. Enephaloid Carcinoma. Anæsthetic Intoxication by Spts. of Turpentine. Report of Amputation in Surgical Cases. Fractures of the Clavicle. Epidemic of Whitlow, etc. Anonymous Writers and Personalities. Report of Surgical Cases, Wounds of Eye, Exophthalmia, Wounds of Face, etc. Wounds of the Small Intestines. Objections to the Use of Air Pumps or Cups in Impaired Vision. Snoring Prevented by Excision of the Uvela. Deaths from Chloroform and Ether. Influence of Climate on Consumption. Observations on the Use of Certain New Remedies. Ovarian Tumors, Rupture of. Extirpation of Cervical Tumors. Cases of Lithotomy. Non-Congenital Talipes Equinus. Quinine and Veratrum Viride in Typhoid Fever. Accidental Castration, with Report of Cases. The Aztecs. Chloride of Soda. Foreign Bodies in the Air Passages. Spermatorrhœa. Yellow Fever. Femoral Exostosis. Utero-abdominal Supporters, Braces, etc. Best Plan of Treating Fractures in Country Practice. Non-Congenital Talipes Varus. Scarlatina. Epidemic Dysentery. Pessaries in Treatment of Prolapsus Uteri. Remarks on the Use of Beverages in Sickness. Consideration of the Relative Value of Lithotrity and Lithotomy. Local Anæsthesia in Surgical Operations. Prolapsus of the Rectum. Non-Congenital Talipes. Local Anæsthesia by Cold. Review of the Mortality Statistics of the Seventh United States Census. Remarks on Diagnosis of Dislocations of the Shoulder Joint. Lithotrity in the Female. Bearded Women. Cases of Lithotomy. American Contributions to Medical Knowledge, editorial. Prevention of Yellow Fever. Fractures of the Scapula. Lecture on the Effects of Intemperance. Review of "Indigenous Races of the Earth." Lecture on Hemorrhoidal Affections. New Principle of Diagnosis in Dislocations of the Shoulder Joint. Treatment of Fractures of Femur Below its Neck. Aneurism of the Ischiatic Artery, Ligation of this Vessel, and, Subsequently of the Primitive Iliac Artery, with Remarks. Fracture of the Neck of the Scapula. Clinical Lecture on Homœopathy. A successful Case of Staphyloraphy. Paralysis of the Upper Extremity Induced Suddenly by a Blow. Clinical Lecture on Rheumatism. Lecture upon Tetanus. Army Diseases. Lecture on Suppuration. Congenital Marks, Deformities, etc. Compound Fracture of Os Femoris Healed in Four Days. Conservative Medicine. Dugas Surgical Pocket Case. Are Crying Babies Necessarily Colicky? Evils of Jostling Infants. Children More Liable to Disease than Adults. Infantile Fevers (2). Errors in Relation to Liver Complaints. Mania a Potu. Ophthalmia Neonatorum. Burns. Remarks on Curability of Inflammation. Mammitis and Mammary Abscesses Treated by Bandaging. Remarks on Some of the Pathological Peculiarities of the Negro Race. Simple Process of Removal of Motes and other Foreign Bodies from Eye. Means by which the Reduction of Strangulated Hernia by Taxis is Materially Facilitated. Remarks on Penetrating Wounds of the Abdomen, with the Suggestion of a Change of Practice in such Cases, read before International Medical Congress, Philadelphia, September, 1876.

It is impossible to attempt, on the present occasion, to enter into an examination of the numerous and voluminous medical writings of Dr. Dugas, nor can a brief synopsis of each of them be presented. This grand man, constantly engaged in an arduous practice of medicine and surgery, published to the profession 127 medical papers, exclusive of a large number of translations of valuable current medical literature. Many of his papers contain original views on the subjects discussed, and some of them record original discoveries made by him.

It would be an injustice to the dead to fail on the present occasion to call attention to the following subjects:

Dr. Dugas was unquestionably the first physician in our State, and I believe in the Union, to call the attention of the profession and municipal authorities to the portability of the poison of yellow fever in railroad cars, and to advocate the breaking of bulk of freight and change of cars from an infected city before reaching one free of the disease. He pointed out the absolute impossibility of thoroughly ventilating box cars by leaving the doors open, and warned municipal authorities of the great danger of the then prevalent practice of depending on this method of railroad sanitary supervision. In demonstration of the justice of this claim I cite his position before the Medical Society of Augusta in 1839, and an article from his pen to be found in the Augusta *Constitutionalist* of February 12th, 1855. It is proper to add in this connection that when the entire medical faculty of Augusta claimed the local origin of yellow fever in our city in the epidemic of 1839, Dr. Dugas was the only physician who dissented from this view. He claimed then, and to the day of his death, that the disease was imported into Augusta by freight and railroad cars from Charleston, S. C.

When the science of surgery knew no certain method for diagnosticating dislocations of the shoulder joint, it was the mind of Dr. Dugas, ever fruitful in investigation and diagnosis, which gave to the profession a method grand in its simplicity and never-failing in certainty, by which the merest tyro in surgery could say with unerring accuracy if or not the shoulder joint was dislocated. Dr. Dugas says of his method: "If the fingers of the injured limb can be placed by the patient or by the surgeon upon the sound shoulder, while the elbow touches the thorax, there can be no dislocation; and if this cannot be done there must be a dislocation. In other words, it is physically impossible to bring the elbow in contact with the sternum or front of the thorax if there be a dislocation; and the inability to do this is proof positive of the existence of dislocation, inasmuch as no other injury of the shoulder joint can induce this inability." This applies to all forms of dislocations of the shoulder joint.

The last medical paper from the pen of the deceased may be justly styled the crowning work of his life, and is destined to revolutionize the practice of surgeons in treating penetrating wounds of the abdomen. While the deceased was the most eminently conservative surgeon I ever knew, he was, when the occasion demanded it, as bold as any surgeon that ever lived. Hence we find him in 1876, at the International Medical Congress at Philadelphia, before the Section on Surgery, where the most brilliant surgeons of the whole world were congregated, saying that peritonitis was not the great cause of mortality from penetrating wounds of the abdomen. He said "the frightful mortality of penetrating wounds of the abdomen, and the rapidity with which the fatal result is brought about, point directly and immediately to septicæmia or blood poisoning, as the only condition at all adequate to such effects; and that the blood poisoning is the almost necessary consequence of the plan of treatment recommended by standard authorities and carried out in daily practice; . . . in all instances of penetrating wounds of the abdomen, in which there may be any suspicion of the existence of effusion of blood or feces into the peritoneal cavity, the surgeon should proceed as follows: 1st. Induce anæsthesia; (2) lay open the abdomen along the linea alba freely enough to make a thorough inspection of the parts contained; (3) ligate all bleeding vessels as far as possible; (4) examine carefully the whole length of the alimentary canal, in order to detect any wounds, and to stitch them. If the intestinal wound be ragged, it should be trimmed down to a straight edge; (5) in gunshot wounds reduce the channels of entrance as well as of exit through the abdominal walls to the form of incised wounds, so that these may be closed and left to adhere by first intention. In some cases it may be found necessary for this purpose to remove the uneven tracks by a double elliptic incision; (6) cleanse the wound and peritoneum of all extraneous materials, and close the abdominal walls with suitable sutures and adhesive plasters; (7) finaly control the peristaltic action with opium, and administer such nourishment as will leave least fecal residuum, as milk, eggs, etc."

In conclusion he adds:

"If the practice now suggested be considered harsh and hazardous, I would ask what can be worse than the authorized plan, which makes death the rule and recovery the rarest exception? Is it not time that we should regard as groundless the fears heretofore entertained with regard to the danger of opening the abdominal cavity? No change of practice in the class of wounds under consideration can make the chances of recovery less than they now are; and I feel confident that by adopting the plan proposed we would so alter the results as to make recovery the rule and death the exception."

Dr. Dugas possessed a mind richly endowed for distinction in any department of medicine, but it was as a surgeon—viewed in the loftiest sense of that term—that his accomplishments shone forth with resplendent eminence. He possessed all the attributes of the great surgeon. He was unusually conversant with the principles of medicine, and therefore fully appreciated the reciprocal connection of its various branches and the dependence of one upon the other. He fully appreciated the conservative and recuperative powers of nature, contended that the genius of surgery consisted in repairing an injury or curing a disease without the use of the knife save as a dernier resort. He had unmeasured contempt for the surgical jobber—dextrous in using the knife, but ignorant of the higher and better parts of the science. He never attempted the so-called brilliant surgical operations which consisted in having an assistant present with watch in hand to proclaim the number of seconds in which he amputated a limb, extracted a calculus or excised a tumor. To the contrary, he was a cautious operator, selecting his subjects with great judgment, and having properly prepared the system of the patient, he with slow and careful strokes of the knife proceeded with the operation with the conscientious conviction that the good of the patient—not the reputation of the operator—should be the sole consideration. He was thoroughly learned in relative anatomy and the fundamental principles of surgery, and made minute and methodical arrangements for every detail of the operation and any emergency which might arise during its progress. His assistants were carefully selected, each one assigned his part, and expected to perform it. After making the necessary preparations, he took the knife and guided it with an unfaltering hand, and step by step carefully proceeded to his work, the results showing the marvelous skill of the surgeon. In surgical diagnosis and prognosis his judgment was almost infallible; manipulations of surgical injuries were made with marked tenderness, and the patient made to feel that he sympathized deeply with his sufferings and soon saw that he was in the hands of a considerate friend and skillful surgeon. He regarded with distrust hazardous operations and untried novelties, and never resorted to either except after weighty deliberation. In his practice he used the simplest dressings—with unusual attention to cleanliness—and avoided showy apparatus and the unseemly display of instruments.

In the practice of medicine he was noted for unmeasured scorn and contempt of the imposter and the polypharmacist. His hospital lectures abound in satire of medical quackery. In this department of medicine he was gifted with a strong and analytical mind in judging of the pathology and natural history of diseases, and every prescription he made was with a definite purpose, guided by a charming simplicity.

As a teacher of medicine, his lectures were remarkably clear, precise and practical; there was an absence of specious theorizing, careless generalizations and lax assertions which made them a blessing to the students. He was singularly devoted to truth. If the truth of any subject discussed lay beyond his comprehension, or if doubts existed in his mind, he was sufficiently great to frankly confess his ignorance of, or doubts upon the subject-matter under consideration. Possessed as he was of a mind of profound erudition and marvelous analytic power, guided by great assiduity of investigation, he carefully winnowed the precious corn of demonstrated facts from the chaff of speculation and doubt. His style of lecturing was marked by unaffected ease of delivery, simplicity of manner, marked perspicuity, and great solicitude to impress his hearers with a knowledge of the themes presented for their instruction. As a consequence he was loved by every student of the college, and by many worshipped as the Gamaliel of Medicine, at whose feet it were an honor to sit and learn.

Dr. Dugas was remarkably kind to, and considerate of the students and younger practitioners of medicine. He was Dean of the Faculty of the Medical College of Georgia for twenty years, and therefore it is needless for me to portray to the two thousand physicians of the South who graduated under him, and the courtesy and kindness with which he met them at the threshold of the profession, and the encouragement with which he sent them out into the world to their high and sacred ministry to suffering and diseased mankind. His love for and kind assistance to meritorious young physicians exceeded that of any man I ever knew. If he saw anything in conduct that was improper, he kindly and considerately admonished the young man of the necessity for a change. Advice or reproof was given with the fatherly tenderness which won the admiration and love of the man reproved, and awakened an ambition to be like unto the fatherly friend. If he called a young physician to his assistance in a surgical operation, he invariably required the patient to pay a proper fee to the assistant as well as the operator.

Dr. Dugas, while an eminent and dearly beloved physician, possessed a mind of rare and varied endowments outside of his profession. Philosophy, science, art, history, etc., had been studied so assiduously and systematically that he was as learned in all of these as in his chosen profession.

Dr. Dugas had many positions of distinction and trust conferred upon him at various periods of his life, among which were the following: He was elected President of the Augusta Medical Society; President of the Medical Association of Georgia; Vice-President of the International Medical Congress, 1876; President of the Board of Trustees of Richmond Academy; President of the Augusta Gas Light Company; President of the Augusta Insurance and Banking Company; Chairman of Committee of City Council to erect a monument to signers of Declaration of Independence, etc. He was elected for a number of years a member of the City Council, and held membership in several scientific and literary associations.

In recognition of his literary and scientific attainments, the University of Georgia, in 1869, conferred on Dr. Dugas the degree of LL. D.

As a citizen, his life was most exemplary. He exhibited great interest in questions affecting the welfare of his city, state and nation, and contributed to the columns of the Augusta newspapers a number of able and thoughtful contributions upon municipal, state and national topics. In all things he was a man of marked independence and fearlessness—not that so-called independence or fearlessness which manifest themselves in acts of turbulence or bravado and indifference to public censure, right or wrong, but his independence was of that character which ornaments chivalrous manhood and dares to brave public opinion and censure when the actor is panoplied with truth and right. He never made merchandise of principles in order that popular approval might signalize his efforts. His acts were determined only after mature deliberation, and from his convictions he was as immovable as the rocks of Gibraltar, unless shown to be in error. An illustration of his manliness and fearlessness is to be found in his opposition to the secession movement of 1861. He entertained no doubt whatever as to the right of the Southern States to secéde from the Union, but was uncompromisingly opposed to it, as he deemed such a step unwise and destructive of the best interest of the South, and was also rather an abandonment of our rights than a manly assertion and vindication of them. His writings evidence profound knowledge of the questions at issue, and his analysis and refutal of arguments in favor of the movement, viewed by the results thereof, show him to have written as though guided by prophetic inspiration. At the time of his writings in opposition to secession, party passion and prejudice were highly inflamed, and only the patriot, with a mind deeply imbued with a sense of right and justice, would have dared to publicly avow them. Opposed as he was to the movement, he, however, like a patriot, cast his fortunes with his State after she had decided to secede from the Union, and did all in his power to advance the interests of the South. His time, talent and money were freely given to his country. He rendered most valuable services to the Confederate government in the medical department. He promptly volunteered his services to the government, and was commissioned surgeon of Walker's command of Georgia troops. In a short time he was commissioned as consulting surgeon to the Confederate hospital in Augusta, where his rare acquirements, experience and conservatism as a surgeon bore rich fruits in the saving of limb and life.

After devoting a long period of life to the relief of human sufferings and the promotion of the best interests of his race, Dr. Dugas did what all wise men propose to themselves, and what but few execute, voluntarily retired from the bustle of life to the quietude of his own home, where he might in the bosom of his family enjoy the society of loved ones, and enjoy to himself time for reflection upon the affairs of life and prepare for its close. His clients heard with regret of his determination to give up his practice, and often the appeals of friendship forced him to respond to calls against which he found it difficult to maintain his resolution. He was one of the founders of the Medical College of Georgia, and continued in its service as the last work of his life. He continued to lecture at the college until 1880, when he resigned his professorship and withdrew from public service. In the full possession of intellectual power, he devoted the remainder of his days to the further cultivation of his mind, and was blessed with contentment and cheerfulness to the day of his death. On the 19th of October, 1884, after a brief illness, death closed his life-work. To the end of his days he retained his wonted intellectual vigor, and at the close had attained to the highest pinnacle of earthly ambition—highly respected, beloved and honored by all who knew him; respected for his marvelous industry and talents; beloved as a most eminent physician, whose skill and tender care had relieved and comforted his fellow-creatures; honored for the possession of those traits of character which constitute the true gentleman in all the walks of life. These form the crown of honor which adorned his brow, and a grateful people lovingly cherish his memory as worthy of everlasting remembrance.

EUGENE FOSTER, M. D.

Augusta, Ga., 1885.

